# The rationale for ophthalmic vigilance during the 2026 Hantavirus outbreak

**DOI:** 10.1038/s41433-026-04848-9

**Published:** 2026-09-09

**Authors:** Aaron T. Zhao, Jordan D. Deaner

**Affiliations:** 1 Perelman School of Medicine, University of Pennsylvania, Philadelphia, PA USA; 2 Retina Service, Wills Eye Hospital, Philadelphia, PA USA; 3 Sidney Kimmel Medical College of Thomas Jefferson University, Philadelphia, PA USA

**Keywords:** Viral infection, Health services

Emerging infectious disease preparedness has traditionally focused on emergency medicine, infectious disease, and critical care specialists. However, the ophthalmologist is not insulated from emerging infectious disease. The 2026 Andes virus outbreak highlights why ophthalmologists should also be incorporated into outbreak surveillance and response. Throughout history, ophthalmologists have played an important role in identifying infectious diseases through their ocular manifestations. Yet, this role depends on a clear understanding of a pathogen’s ophthalmic phenotype. Despite growing attention surrounding the current Andes virus outbreak, the ocular manifestations of this pathogen remain largely undefined. This knowledge gap presents both an opportunity and a responsibility for ophthalmologists to systematically characterise ocular involvement and contribute to emerging infectious disease surveillance. Herein, we review the available evidence regarding ocular involvement in hantavirus infection and contend that ophthalmologists have a critical role in defining the ocular phenotype of Andes virus during the current outbreak.

The Andes strain of Hantavirus was recently documented aboard an international vessel. In April 2026, passengers and crew aboard the MV Hondius, began developing severe respiratory illness during an itinerary across the South Atlantic. The World Health Organization was notified on May 2, 2026, and Andes virus was confirmed as the causative pathogen on May 5 [1]. As of late-May 2026, 13 confirmed or probable cases and 3 deaths had been reported across multiple countries, with the Centers for Disease Control and Prevention (CDC) monitoring several individuals across multiple US states [2]. Although the overall public health risk in the United States remains low, the CDC has issued a Health Alert Network advisory emphasising heightened clinical vigilance.

Hantaviruses (family Hantaviridae) are enveloped, single-stranded, negative-sense RNA viruses transmitted primarily through aerosolisation of infected rodent excrement, causing two principal syndromes: hantavirus pulmonary syndrome (HPS) from New World hantaviruses including Sin Nombre and Andes viruses, and haemorrhagic fever with renal syndrome (HFRS) from Old World hantaviruses such as Puumala, Hantaan, and Seoul viruses. Andes virus is the only hantavirus with recently confirmed person-to-person transmission, typically via respiratory secretions during prolonged close contact with a symptomatic individual [3].

The ophthalmic literature on hantavirus is dominated by Puumala virus, which causes nephropathia epidemica (NE), a generally mild to moderate, renal-predominant form of HFRS. In the largest prospective ophthalmic series to date, Hautala et al. examined 92 eyes of 46 serologically confirmed NE patients and found that 70% reported ocular symptoms during acute illness, with decreased IOP in 88%, reduced visual acuity in 87%, conjunctival chemosis in 87%, crystalline lens thickening in 82%, a myopic shift in 78%, anterior chamber shallowing in 64%, and shortened vitreous length in 52% [4]. All anterior segment changes resolved fully with systemic recovery. Transient myopia is the hallmark finding, occasionally exceeding −4.0 diopters [5]. External findings include periorbital oedema, conjunctival injection, and subconjunctival haemorrhage [4–6]. Posterior findings are less frequent but include retinal haemorrhages, macular oedema, retinal whitening near the optic disc, and a self-limiting posterior necrotising retinitis, with neuro-ophthalmic manifestations possible when CNS complications such as hypophyseal haemorrhage occur [5].

Although ocular manifestations of Puumala strain of hantavirus infection are well described, virtually no ophthalmic data exist for Andes virus, the pathogen responsible for the current outbreak. This distinction is critical because existing ophthalmic knowledge is derived from a different virus, a different clinical syndrome, and a substantially different severity spectrum. What we do know is that in contrast to the Puumala virus, the systemic manifestations of the Andes virus causes HPS, a fulminant cardiopulmonary illness in which patients progress rapidly to respiratory failure, mechanical ventilation, and in severe cases extracorporeal support [7]. To date, no ophthalmic evaluation of HPS patients has been published.

This gap in knowledge has practical consequences. Clinically, this means that the pattern of presentation that ophthalmologists may be tasked to recognise is extrapolated from a population that differs in virus, syndrome severity, and clinical state. The existing literature on the Puumala virus may be used to prompt suspicion (i.e., a febrile patient with bilateral unexplained myopic shift should raise the possibility of hantavirus), but it cannot yet tell ophthalmologists what Andes virus ocular disease looks like beyond that. The current outbreak represents not only a clinical challenge but also a scientific opportunity. For ophthalmologists, this means that every HPS patient managed at a US referral centre during the current outbreak who is well enough to undergo ophthalmic evaluation represents an opportunity to generate evidence that does not yet exist. A standardised assessment, documented and reported in the literature, would begin to answer a question that the existing literature cannot.

Importantly, ophthalmologists should examine patients with known or suspected hantavirus using correct transmission-based precautions. Unlike other hantaviruses, Andes virus has documented person-to-person transmission via respiratory secretions, a characteristic with no known analogue among other hantaviruses. For ophthalmologists, this distinction is particularly relevant because slit-lamp examination requires prolonged face-to-face proximity. Consequently, infection-control preparedness should be considered. The CDC recommends airborne infection isolation with gown, gloves, eye protection, and an N95 or higher-level respirator for any patient with confirmed or suspected infection [2].

Historically, ophthalmologists have contributed immensely to the recognition and characterisation of systemic infectious diseases through their ocular manifestations. From syphilis and tuberculosis to more recent outbreaks involving Zika virus, Mpox, and COVID-19, ocular findings have occasionally provided diagnostic clues, insight into disease pathogenesis, and opportunities for longitudinal monitoring. The Andes virus should be viewed within this broader framework.

The 2026 Andes virus outbreak carries with it a powerful lesson. We as ophthalmologists are not peripheral observers during emerging infectious disease events. We contribute to surveillance, diagnosis, characterisation of novel ocular manifestations, and implementation of appropriate infection-control practices. As the outbreak evolves, systematic ophthalmic evaluation of affected patients may help close a major knowledge gap regarding the ocular manifestations of the Hantavirus Andes strain infection.

## Data Availability

This viewpoint article does not contain original data. All data supporting the arguments presented are available in the published studies cited in the reference list.
